# Applied and Translational Genomics for Human Genetics and Clinical Science

**DOI:** 10.3389/fbioe.2014.00011

**Published:** 2014-05-09

**Authors:** Dae-Won Kim

**Affiliations:** ^1^Systems Biology Team, Center for Immunity and Pathology, Korea National Institute of Health, Korea Centers for Disease Control and Prevention, Cheongwon-gun, South Korea

**Keywords:** computational genomics, translational bioinformatics, systems biology for human genetics, next-generation sequencing (NGS), personalized medicine

Translational bioinformatics is an emerging field of study that addresses the computational challenges encountered in biological and clinical research as well as in the analysis and interpretation of the data generated from it. Applied computation genomics, as part of the Springer Series on Translational Bioinformatics, thoroughly discusses the most relevant issues in the development of novel techniques for the integration of human genetic, biological, and clinical data. This book also provides an example of theories and research being practically applied to inform translational medical research in clinical diagnosis. This book covers numerous experimental and computational methods related to statistical development and their applications in the field of human genomics, including candidate gene mapping, linkage analysis, population-based, genome-wide association, exon sequencing, and whole genome sequencing analysis.

This book consists of 10 chapters. The first chapter focuses on an overview of the current human genome science. It reviews the history of using machine-learning algorithms for studies on disease prediction and provides highlights for the other nine chapters, which have been collected in this book. Chapter 2 provides a broad overview of the most important concepts in genetic epidemiology. In this chapter, the authors provide a precise definition for complex traits and a thorough introduction to genetic epidemiology as a tool for understanding the role of genetic factors. In addition, the essential study designs used to accomplish this goal, including family, twin, adoption, and migration studies, are summarized. Chapter 3 focuses on integrated linkage analysis and its results in the design, execution, and interpretation of whole genome or whole exome sequencing studies. It includes experiments, knowledge, and specific example data. This chapter also presents new statistical algorithms to identify rare variants in pedigree settings for both qualitative and quantitative traits. Chapter 4 briefly reviews the methods used to combine functional genomic data to detect complex diseases. Chapter 4 examines the progress of research on a specific rare disorder, nasopharyngeal carcinoma (NPC). Dr. Jorgensen et al. conducted a thorough review of all candidate genes related to NPC and explained the findings of two genome-wide association studies (GWAS), one by a research group in Taiwan and the other by a group located in Guangzhou, China. This section also emphasizes the need for an effective system to handle future community genome arrays (CGAs) and GWAS, to maximize their information content, and make the best use of the limited number of available NPC study populations. Chapter 5 introduces genetic mapping of quantitative trait loci (QTL), offering an efficient and powerful method to find putative regulatory regions and to discover novel functional impact of genetic variants. Here, the authors describe recent studies on QTL mapping of complex traits, including DNA methylation, gene expression, and protein expression as well as metabolites. The authors summarize several exciting findings from eQTL analysis in various human post-mortem brain tissues based on publications, which appeared between 2007 and 2012. Chapter 6 explains the pivotal role of genetic haplotypes in gene prediction. The authors define a haplotype with the theoretical background of haplotype analysis and provide their perspective on the development of a new likelihood-based method that make predictive models using genotypes data derived from unrelated individuals. Finally, it provides current technologies and software for a clear genetic interpretation focused on the HLA region. Chapter 7 discusses the overall knowledge on whole exome sequencing. The authors comment on current analytical challenges such as image processing, base-calling, and short read mapping, and introduce the main statistical approaches that can be used to discover rate variant and copy number variations. Through this chapter, the readers could grasp analytical concepts of biological big data analysis related to sequencing alignment and variant calling as well as data management. Chapter 8 introduces the strategies and mathematical algorithms for analyzing rare variants such as weighted sum association method (WSM), sequence kernel association test (SKAT), and odds ratio weighted sum statistic (ORWSS). Chapter 9 introduces gene duplication and functional consequences, and focuses on copy number variation as well as segmental duplications and gene essentiality in duplicated genes, transcriptional, epigenetic divergence following gene duplication, and environmental adaptation. In this chapter, the authors use their own experimental material as an example and explain how to interpret the correlation between copy number variations and complex traits in humans, such as psychiatric disorders. The final chapter 10 strives to serve as a bridge to connect the content provided by the previous chapters, defines translational medicine, and explains how next-generation sequencing (NGS) transformed current genetic research especially in the area of cancer and mental health. At the end of this chapter, the authors discuss the limitations of GWAS as well as the rise of NGS and present applications of NGS to the research on human complex diseases and personalized medicine.

Ultimately, this book provides an insightful view of computational genomics knowledge, techniques, and tools used in an interdisciplinary field. Overall, this volume strives to provide a very comprehensive review of scientific issues related to human genetics and clinical research technologies. Most of the authors are well known in their research fields. The chapters are timely, well structured, and informative. This book could be considered as great reading material for graduate courses in bioinformatics and functional genomics, but, as suggested by the subtitle, could serve as a basis for selected case studies.

## Author Contributions

Dae-Won Kim: conception, drafting, and final approval of the manuscript.

## Conflict of Interest Statement

The author declares that the research was conducted in the absence of any commercial or financial relationships that could be construed as a potential conflict of interest.

